# Coxiellosis in Dogs—A Hitherto Masked Zoonosis in India: An Insight From Seromolecular Investigation and Risk Factor Analysis

**DOI:** 10.1155/cjid/8642619

**Published:** 2025-01-20

**Authors:** Valil Kunjukunju Vinod, Satyaveer Singh Malik, M. S. Sivaprasad, Chinmay Malik, Neha Parmar, Karikalan Mathesh, Brijesh Kumar, Ujjwal Kumar De, E. S. Sanjumon, Jess Vergis, Sukhadeo Baliram Barbuddhe, Deepak Bhiwa Rawool

**Affiliations:** ^1^Division of Veterinary Public Health, ICAR-Indian Veterinary Research Institute, Izatnagar, Uttar Pradesh, India; ^2^Pre-Med Scholar, College of Health Solutions, Arizona State University, 1151 S Forest Ave, Tempe, Arizona, USA; ^3^Centre for Wildlife, ICAR-Indian Veterinary Research Institute, Izatnagar, Uttar Pradesh, India; ^4^Division of Animal Reproduction, ICAR-Indian Veterinary Research Institute, Izatnagar, Uttar Pradesh, India; ^5^Division of Medicine, ICAR-Indian Veterinary Research Institute, Izatnagar, Uttar Pradesh, India; ^6^Department of Veterinary Public Health, College of Veterinary and Animal Sciences, Kerala Veterinary and Animal Sciences University, Pookode, Kerala, India; ^7^Meat Microbiology, ICAR-National Meat Research Institute, Hyderabad, Telangana, India

**Keywords:** canine coxiellosis, companion animals, IFAT, India, risk factor analysis

## Abstract

*Coxiellaburnetii* is an airborne bacterial zoonotic pathogen that causes Q fever/coxiellosis in humans and animals. Although dogs are suspected of transmitting Q fever to humans in past outbreaks, the prevalence of *C. burnetii* in the Indian dog population and risk factors for infection remain unknown. In this study, 452 dogs from pet clinics in three Indian states were screened for coxiellosis using molecular (Trans-PCR, Com 1-PCR) and serological (IFAT) tests. *C. burnetii* DNA was detected in 0.44% of blood samples using Trans-PCR, and pathogen-specific antibodies were found in 4.20% of sera using IFAT. Contact with stray dogs and ownership by farmers were identified as risk factors for canine coxiellosis. This study appears to be the first systematic assessment of coxiellosis and associated risk factors among dogs in India. A large-scale assessment of canine coxiellosis and its risk factors is warranted among pets and high-risk occupational groups in India.

**Summary**
• This study appears to be the first systematic assessment of coxiellosis among dogs and risk factors in India.
• Occurrence of *Coxiella burnetii* in dogs was found to be 4.20% in IFAT.
• *C. burnetii* DNA was detected from 0.44% of blood samples from dogs.
• Identified risk factors were contact with stray dogs and ownership by farmers.
• Point-of-care diagnostics are needed for extensive screening of canine coxiellosis.

## 1. Introduction

Coxiellosis, or Q fever, is an airborne zoonotic infection caused by the obligate intracellular bacterium *Coxiella burnetii*, and it has been reported in most countries, except New Zealand, Iceland, French Polynesia, and Norway [1]. It is a small Gram-negative bacterium (0.4–1 μm in length and 0.2–0.4 μm in width), with a doubling time of approximately 20–45 h in cell culture [2]. This highly infectious and contagious pathogen [3] is capable of causing the disease with even a single cell [4]. The Type I, II, and IV secretion systems, along with lipopolysaccharide (LPS), are involved in disease causation by *C. burnetii* [5, 6]. *C. burnetii* transitions from a virulent Phase I form to an avirulent Phase II form, characterized by LPS truncation [6] when passaged in tissue culture or embryonated eggs. It has been reported that *C. burnetii* in Phase I primarily reacts with late-stage sera, while organisms in Phase II react with both early (< 20 days) and late (> 20 days) sera derived from infected guinea pigs [7]. For this reason, commercial indirect fluorescent antibody test (IFAT) slides are typically coated with separate Phase I and Phase II antigens in each well.

In humans, although Q fever is a rare and sporadic disease, it causes significant morbidity with sparse mortality and has widely variable clinical symptoms and manifestations [2]. Domestic ruminants are the main reservoirs of *C. burnetii*. However, dogs, cats, birds, and ticks also play a role in its transmission. Dogs and cats play a minor role in transmitting *C. burnetii* [8]. Contrary to ruminants, the documented cases of human Q fever acquired from dogs appear scanty and include only three brief reports. However, children playing with pets were reported to be at significant risk of exposure to *C. burnetii* [9]. The consequences of Q fever can be severe in ignorant pet owners/breeders, unvaccinated veterinarians, and para-veterinary staff.

The actual status of *C. burnetii* infection in pet dogs, along with its associated risk factors and disease dynamics, remains largely unknown in India, mainly because of the lack of scientific reports on Q fever in humans linked to dogs. Additionally, poor disease surveillance, a general lack of awareness, and clinical curiosity among veterinarians and healthcare professionals might have contributed to the already neglected and underreported status of coxiellosis in India. Additionally, infected dogs exhibit no apparent symptoms, making them a potential risk to both human and livestock health [9]. Assuming the low carriage rates of *C. burnetii* in over 19.41 million pet dogs and 35 million stray dogs in India [10], it is logical to speculate that the disease may be grossly underdiagnosed and underreported in dogs due to the absence of specific disease symptoms. Therefore, a realistic assessment of the potential zoonotic risk of *C. burnetii* infection from dogs to humans within the veterinary community and the broader dog-owning communities requires robust population-based studies to examine past exposures or current infection status in dogs, which is eluding so far. Thus, the present investigation aimed at screening coxiellosis among pet dogs using molecular and serological tests. Further, the risk factors contributing to *C. burnetii* infection among dogs were assessed through a self-designed questionnaire.

## 2. Materials and Methods

The study was carried out during the period from August 2020 to October 2021 in the Comparative Pathology and Biomedicine (CPB) laboratory, Division of Veterinary Public Health (VPH), ICAR-IVRI, Izatnagar, Bareilly, Uttar Pradesh (UP).

A total of 452 dogs from four Indian cities (Bareilly, Bhopal, Kochi, and Thiruvananthapuram) across three states (UP, Madhya Pradesh (MP), and Kerala) were screened for coxiellosis by molecular and serological assays. The blood (*n* = 452) and serum (*n* = 452) samples were collected from dogs presented for clinical examination or routine health check-ups. In addition, 37 vaginal swabs and 35 uterine tissue samples were also collected and screened for *C. burnetii* by molecular assays.

Dogs under four months of age were excluded to avoid detection of maternal antibodies. Sera from dogs undergoing antibiotic treatment at the time of sampling were also excluded. All dogs were handled ethically.

### 2.1. Sampling Location

**Figure 1:**
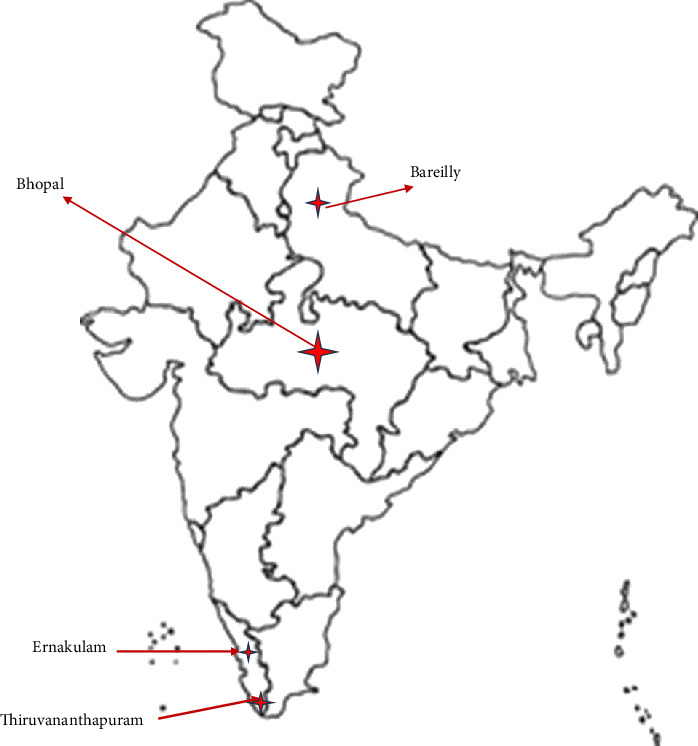
Locations of sample collection in this study.

The clinical samples were collected from dogs presented at Referral Veterinary Polyclinic (RVP), ICAR-IVRI, Izatnagar, Bareilly, UP; Pet clinics in Kochi, Kerala; Government Referral Veterinary Hospital, Kudappanakunnu, Trivandrum; District Veterinary Center, Trivandrum, Kerala; and Veterinary dispensaries in Bhopal, MP. Of the 452 dogs sampled, 206 were from UP, 181 from Kerala (106 samples from Ernakulam and 75 from Trivandrum), and 65 from MP (Figure 1).

### 2.2. Epidemiological Data Collection

The data on breed, sex, age, habitat, housing, hygiene-related factors, and exposure to stray or other domestic animals were collected from pet owners using a structured questionnaire. The clinician's assessment, clinical biochemistry, and hematological parameters were also recorded. The sampling method used in this study was convenience sampling, as remote areas were practically difficult to access. Most of the previously published studies on coxiellosis in dogs also followed convenience sampling [11, 12].

### 2.3. Blood Sampling, DNA Extraction, and Serum Separation

The clinical samples (blood, serum, vaginal swabs, and uterine tissue) were collected aseptically. Briefly, aliquots of blood samples (5 mL each) were collected aseptically in a 5 mL spray-coated K2EDTA tube (Vacutainer, Becton Dickinson, Franklin Lakes, USA) for blood analysis and into 5 mL tubes of SST II Advance (Vacutainer, Becton Dickinson, Franklin Lakes, USA) for serum separation.

The vaginal swabs (HiMedia Laboratories Pvt. Ltd., Mumbai) were collected from pregnant and postparturient (1–4 weeks after parturition) dogs using sterile swabs after disinfection of the vulva. All swabs were inserted in sterile screw-capped polypropylene tubes (HiMedia) containing 1 mL of sterile phosphate buffer saline (PBS). The uterine tissue samples were collected in a sterile sample container with a lid (HiMedia) containing sterile PBS. The collected samples were transported under cold chain at 4°C.

The clot activator tubes containing blood samples were centrifuged at 2500 × g for 10 min for serum separation. For DNA extraction from tissues, 25 mg of tissue was sliced aseptically using a scalpel blade and homogenized in a sterile Petri dish. The vaginal swabs in microcentrifuge tubes containing 200 μL of sterile PBS were vortexed, and 200 μL of the suspension was used for DNA extraction [13].

The DNA from blood, serum, vaginal swabs, and tissue samples was extracted using commercial DNA extraction kits (Qiagen, USA) following the manufacturer's instructions. The concentration and purity of the eluted DNA were determined by measuring the optical density at both 260 nm and 280 nm using a Bio-Spectrometer (Eppendorf). The purified DNA was stored at −20°C for subsequent analysis.

### 2.4. Serological Investigation

The sera samples collected from dogs were tested for antibodies against *C. burnetii* using a commercial canine IgG-IFAT kit (Fuller, Fullerton, USA) as per the manufacturer's protocol. Accordingly, all the test sera were diluted to 1:16 using PBS containing goat serum and pipetted into the separate wells of a 12-well slide supplied with the kit. The processed slide was examined under fluorescent microscope (Nikon Eclipse NI, Japan) at 400X magnification. The fluorescence intensity and appearance of each well were compared with the positive and negative control wells for assessment. When viewed through microscopic optics, the phase II antigen dot appeared on the left and phase I on the right side of the field. Bright staining (at least 1+) of the elementary bodies against a background of red counterstained yolk sac material indicated a positive reaction.

The test fields were compared with positive and negative controls for particle size, appearance, and density. A reactivity pattern differing from that of the positive control was considered nonspecific. IgG titers of 1:16 or higher against phase II antigens indicated seropositivity at an unspecified time point. A negative reaction against *C. burnetii* antibody was recorded when the fluorescence intensity from a well at 1:16 screening dilution was similar to that of the negative control. The photographs of the slides (positive control, negative control, and test sample) were taken using NIS-Elements Imaging software (Nikon).

### 2.5. Molecular Testing

All the clinical samples were screened for the pathogen DNA by Trans (targeting multicopy gene *trans* of *C. burnetii*) [14] and Com-1 (targeting single-copy gene com-1 of *C. burnetii*) [15] PCR assays. The details of the primers used and the PCR conditions are provided in Table 1.

### 2.6. Risk Factor Analysis

Risk factor analysis was conducted to identify risk factors for the seropositivity against *C. burnetii*. Data for the analysis of risk factors were obtained from the dog owners through a self-designed questionnaire (Appendix 1) about themselves, their pets, managemental practices, and the type of housing provided to them. The clinical parameters of dogs were recorded individually at our end. The target variable (IFAT) was dichotomized (positive/negative). Each potential risk factor was studied for its association, if any, with the development of disease, and its significance level was calculated using the chi-square test.

### 2.7. Statistical Analysis

The GraphPad Prism software (GraphPad Prism 8.0.2) was employed to evaluate statistical significance. The chi-square test was used to determine the association between risk factors and the seropositive results of Q fever.

## 3. Results

The current study reported an overall positivity rate of 0.44% using Trans-PCR and 4.20% using IFAT for coxiellosis. None of the blood samples tested positive for Com1-PCR. Furthermore, none of the serum, vaginal swabs, or uterine tissue samples were positive for *C. burnetii* DNA in either the Trans-PCR or Com1-PCR assays. Overall seropositivity for coxiellosis among dogs was recorded as 2.91% (6/206) in Bareilly (UP), 2.83% (3/106) in Ernakulam (Kerala), 6.67% (5/75) in Trivandrum (Kerala), and 7.69% (5/65) in Bhopal (MP) (Tables 2 and 3). No correlation between IgG-IFAT and PCR assay could be established by the chi-square test using GraphPad Prism 8.0.2.

Risk analysis based on the responses obtained from 372 dog owners (Table 4) indicated that male dogs exhibited 2.165 times higher risk of contracting *C. burnetii* infection compared to female dogs; however, this difference was not statistically significant (*p* > 0.05). Nonetheless, dogs that had contact with stray dogs were found to have a significantly higher risk (4.064 times; *p* < 0.0041) of contracting *C. burnetii* compared to those without such contact.

**Figure 2:**
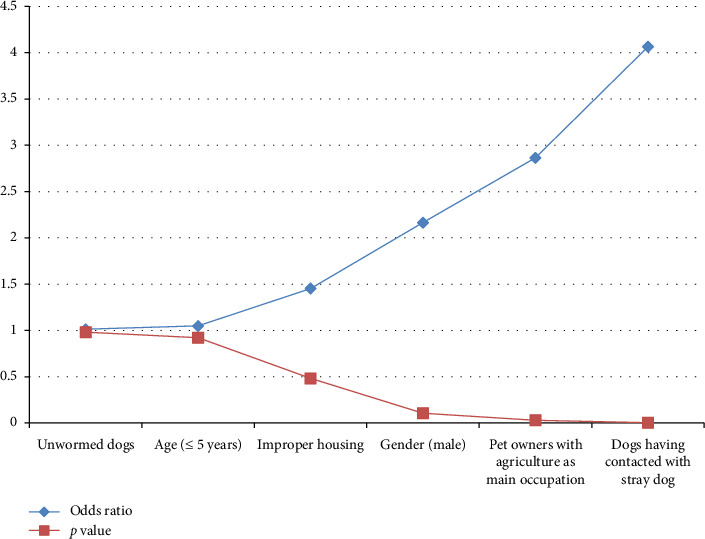
Risk factors associated with anti-*C. burnetii* antibodies in dogs in selected cities of India.

Noncaged and male dogs appeared to be more susceptible to coxiellosis compared to caged and female dogs, although this difference was not statistically significant. Nevertheless, dogs owned by individuals involved in agriculture and animal husbandry exhibited a significantly higher risk of infection (2.85 times; *p* < 0.0302) compared to those owned by people in other occupations. The results obtained are illustrated through a graphical representation (Figure 2), and the statistical analysis is provided as supporting data.

## 4. Discussion

Dogs have been identified as a potential source of *C. burnetii* infection for their owners and other pets, with studies highlighting the role of stray dogs in spreading the infection [16]. Consequently, dogs are considered reservoirs, secondary reservoirs or sentinels, and potential vectors [17, 18] for transmitting *C. burnetii* to humans. The definitive diagnosis of coxiellosis requires confirmation through both molecular and serological assays. However, previous studies have primarily relied on either serological or molecular tests, often involving small sample sizes. Moreover, a comprehensive investigation to assess the prevalence of coxiellosis in the dog population in India has yet to be conducted. Therefore, in the present study, dogs from selected cities across three different Indian states were screened for coxiellosis using both molecular and serological tests.

*C. burnetii* DNA was detected in 0.44% of the blood samples from domestic dogs tested using Trans-PCR. The results could not be compared to findings from India, as no similar studies have been published to date. The positivity rate observed in this study was slightly higher than that reported in some other regions of the world, where rates were recorded as 0% in countries such as China [19], Italy [20], Australia [11], Pakistan [21], and Egypt [22]. However, it was lower than the positivity levels reported elsewhere, such as 7% in the Netherlands [17, 18], 8% in Cambodia [23], 10% in Zambia [24], 11% [25] in Iran, and 33% in Malaysia [16]. The variations in positivity rates across these reports may be attributed to differences in the screened dog populations, housing environments, coxiellosis/Q fever epidemiology in the sampled regions, the sensitivity and specificity of the molecular tests used, and the target genes selected. For example, the dog population with a 7% positivity rate for coxiellosis was screened shortly after a major Q fever outbreak in the Netherlands [17]. Similarly, most of the dogs from Cambodia that tested positive in PCR were from areas with limited access to proper veterinary care, including antiparasitic treatments, spaying/neutering, and vaccinations [23]. Likewise, the dogs sampled from Zambia were from an area where 45% of the rodents tested positive for *C. burnetii* DNA [24] indirectly indicating a high pathogen load in the sampled environment. Similarly, tick infestation could have led to a higher positivity of dogs in Iran for coxiellosis, which was as high as 11% [25]. In contrast, studies conducted in the northern states of India have ruled out the involvement of ticks as vectors for *C. burnetii*, while a low positivity rate (12/525) for *C. burnetii* in ticks was reported in the northeastern states [26].

In our study, none of the tested clinical samples were positive for coxiellosis in the Com1-PCR, consistent with the observation of a similar nature from other workers [11, 19]. In the present investigation, two dogs that previously tested positive in the Trans-PCR assay were found negative for anti-*C. burnetii* antibodies in the canine IgG-IFAT kit, indicating an early stage of the infection before seroconversion.

The nondetection of *C. burnetii* in 37 vaginal swabs of dogs by PCR assays observed in our study corroborates with the observations of similar nature made by other workers [11]. The DNA could not be detected in vaginal swabs from two dogs that gave positive Trans-PCR results in blood samples. It is worth noting that shedding of the pathogen in companion animals like dogs and cats has been considered rare, even after higher exposure to this pathogen [11]. Our findings align with reports from studies on other animal species, such as cattle, where intermittent or even no shedding of the bacteria was observed in vaginal discharges despite testing positive for *C. burnetii* infection in molecular assays [27, 28].

Similarly, none of the 35 uterine tissue samples tested positive for the pathogen in molecular assays. This finding is consistent with previous studies, where uterine or placental tissues and endometrial swabs also yielded no positive PCR results for *C. burnetii* DNA [8, 22]. Likewise, a study conducted in Australia found that none of the reproductive tissues or vaginal/preputial swabs tested positive in qPCR assays employing *IS1111* and *com1* genes [11]. These findings may be attributed either to the absence or low concentration of the pathogen in the tested samples. However, they differ from studies in which *Coxiella* DNA was detected in 7% of placentas from parturient female dogs following the 2007–2010 Q fever outbreak in the Netherlands [17].

The role of *C. burnetii* in causing reproductive abnormalities in dogs and cats is still obscure or not fully understood [29] and warrants further exploration. The current understanding of the pathogenesis of *C. burnetii* infection in companion animals remains incomplete, as it is primarily based on data extrapolated from human coxiellosis outbreaks associated with cats. This leaves significant gaps in knowledge regarding the development and progression of natural infections in companion animals, host seroconversion, and bacterial shedding following exposure to *C. burnetii*. Therefore, clear evidence of pathogen shedding in dogs that test positive for coxiellosis through molecular and/or serological tests is essential to define and quantify the potential risk to public health.

In this study, 4.20% (19/452) of serum/plasma samples of dogs tested positive for anti-*Coxiella* IgG antibodies using a canine IgG-IFAT kit. The seropositivity observed in dogs sampled from northern, central, and southern states of India was 2.91%, 7.69%, and 4.41%, respectively. The present findings were comparable to the seropositivity reported as 5.2% in French Guyana [30], 5.5% in Iraq [31], 1.9%–6.5% in Australia [32], 2.9% in South Korea [33], and 8.3% in Italy [12].

In general, the data about the dissemination of coxiellosis in companion animals appear either scanty or minimal [12]. In the global context, the reported level of seropositivity in dogs for coxiellosis has ranged from a low of 0% in Italy [34] and Canada [35] and to a higher level of 9.8% in France, 11.6% in Senegal [30], 13.8% in Iraq [36], 14.8% in Zimbabwe [37], 15% in Japan [38], and 26.1% in Australia [11]. According to the available reports, the seroprevalence of *C. burnetii* infection, particularly in companion animals, varies with geography, land-use factors, and shared environmental sources [11]. So, it is assumed that, like in cattle, environmental factors also play a role in the frequency of exposure to this pathogen [39] in dogs. A recent geostatistical regression-based study in Ethiopia also showed that seropositivity against *C. burnetii* in cattle varied across geographical locations and was associated with environmental risk factors [40]. Similarly, the seroprevalence of coxiellosis in white-tailed deer ranged widely from 0% to 32.0% across New York, U.S.A [41].

In the present study, all 19 sera of dogs that tested positive for coxiellosis in the canine IgG-IFA test kit reacted against the phase II antigen of *C. burnetii*, suggesting that the infection in these dogs occurred at an undetermined time. This finding agreed with a similar observation reported earlier [12]. In many studies, canine samples tended to be positive for either phase I or phase II antigen, with a small number of samples showing positivity for both [11] as the process of seroconversion is not well characterized in these animals, and seropositivity to either or both antigenic phases of this pathogen has been shown to vary between species [35].

Comparatively lower seropositivity observed among dogs from Bareilly, UP, and Kochi and Trivandrum in Kerala can be attributed to the low endemicity of coxiellosis in sampling areas. The seroprevalence of coxiellosis in cattle and goats in the Bareilly region has been reported as 5.5% and 6.08%, respectively [42], while the seroprevalence in cattle in Kerala was 4.5%. The relatively higher seropositivity (7.69%) for coxiellosis observed among dogs in Central India requires further evaluation and validation in a larger dog population, focusing on identifying the factors contributing to the increased occurrence of the pathogen in this region. Notably, a seroprevalence study conducted on a small sample of cattle in MP found that 28.5% tested positive for coxiellosis by ELISA [43]. Therefore, the higher seroprevalence of coxiellosis in the canine population of Bhopal (MP) may be linked to the widespread presence of the organism in other animal species. Studies have shown that climatic conditions and landscape patterns influence the seroprevalence of coxiellosis in small ruminants across both narrow and broad geographic regions [44].

In this study, the potential risk factors for coxiellosis in dogs were evaluated based on the detailed information on sampled dogs, their owners, clinician's health assessment, clinical biochemistry, hematological parameters, type of housing, and factors related to hygiene and sanitation.

The study found that male dogs had a higher, though not statistically significant (*p* > 0.1542 at 95% CI), risk of contracting *C. burnetii* infection compared to female dogs. This observation is consistent with findings from previously reported studies [16, 45]. With no significant differences observed in the overall behavior of male and female dogs, the higher likelihood of male dogs contracting *C. burnetii* infection may be attributed to their more territorial, active, and easily distracted nature, which could increase their chances of exposure to other potentially infected dogs or domestic ruminants [16]. Another plausible reason for the lower seropositivity of female dogs observed in our study could be the reported sensitivity of *C. burnetii* to progesterone, which has been shown to inhibit its replication in cell-free cultures [46] directly.

It was not surprising that the dogs having contact with stray dogs/cats were found to have a significantly higher risk (*p* < 0.0041 at 95% CI) of contracting *C. burnetii* infection than those without contact. These findings can be explained in light of the earlier reports wherein stray dogs exhibited high seropositivity against *C. burnetii*. This may be attributed to malnutrition, compromised immunity, and exposure to a susceptible environment, as observed in feral dogs [25]. Recent research has also shown that stray dogs may serve as a source of dissemination of *Coxiella* infection to humans and other naive dogs [16].

We found the risk of contracting *C. burnetii* infection among noncaged dogs compared to caged ones to be nonsignificant (*p* > 0.05 at 95% CI). However, outdoor housing of dogs was reported to enhance the chances of getting coxiellosis from stray dogs or other domestic livestock [25] and underlines the importance of proper housing for pet dogs, especially in endemic areas, to protect them from contracting coxiellosis.

We observed a significantly (*p* < 0.0302 at 95% CI) higher seropositivity against *C. burnetii* among dogs owned by persons engaged in agriculture and animal husbandry sectors than those not involved in such activities. This finding aligns with similar findings from other researchers that the dogs of pet owners who had worked closely with other animals had a positive correlation with seropositivity to *C. burnetii*. Dogs living in rural and agricultural areas appear to face an increased risk of seropositivity to *C. burnetii*, possibly due to more frequent exposure to environmental contaminations from infected primary reservoirs, such as cattle, sheep, goats, and ruminants [11].

In this study, exposure to *C. burnetii* in dogs was confirmed by both molecular and serological methods. However, the role of dogs as potential reservoirs or carriers for *C. burnetii* needs a detailed investigation in a larger canine population from diverse geographic areas.

## 5. Conclusions

This investigation aimed to assess the status of coxiellosis among healthy and suspected/diseased dogs of diverse geographical areas in India. Appearing to be a study first of its kind in India, the occurrence of coxiellosis in dogs from three states, i.e., UP, Kerala, and MP, was established by serological and molecular screening. We could not describe any definitive source/sources of *Coxiella* infection among the screened dogs. Nevertheless, a common/shared source of infection can be a plausible reason for this geographical variation in seroprevalence. Dogs kept indoors could be exposed to potential sources/reservoirs of *C. burnetii*, thereby acting as a source of infection to human beings. Therefore, in contrast to the earlier studies that disregarded the role of dogs in transmitting Q fever, we believe that studies on a larger sample size from different geographic locations in India need to be conducted. It is also worth mentioning that, currently, IFAT is the only commercially available serodiagnostic tool for coxiellosis in dogs, while dedicated commercial ELISA kits for dogs are elusive. Furthermore, these commercial test kits are costly for mass-screening dogs for coxiellosis. The lack of user-friendly diagnostics adds to the existing pile of stumbling blocks for conducting seroprevalence studies on canine coxiellosis in resource-limited countries. Hence, economical and point-of-care serodiagnostic assays are a priority in the extensive screening of dogs for coxiellosis.

## Figures and Tables

**Table 1:** Oligonucleotides and PCR conditions employed for detection of *C. burnetii* in clinical samples.

Target gene	Primer	Primer sequence (5′—3′)	Product size (bp)	Reference(s)	PCR conditions (°C/seconds)			No. of PCR cycles
					Denaturation	Annealing	Extension	
*IS1111*	trans 1	TAT GTA TCC ACC GTA GCC AGT C	687	Berri et al. 2000	95/30	52/30	72/60	35
	trans 2	CCC AAC AAC ACC TCC TTA TTC						

*com1*	com1	AGT AGA AGC ATC CCA AGC ATT G	501	Zhang et al. 1998	95/30	63/60	72/90	30
	com2	TGC CTG CTA GCT GTA ACG ATT G						

**Table 2:** Area-wise and sample-wise details of dogs tested positive for anti-*C. burnetii* antibodies.

Sl No.	Place of sample collection	Case ID	Age (years)	Sex	Occupation of the owner	Presence of hard ticks (Y/N)	Contact with stray dogs	Type of housing
1	Bareilly, Uttar Pradesh	VSD-77	≤ 5	Male	A	Y	Y	U
2		VSD-78	≤ 5	Female	A	N	N	U
3		VSD-1087	> 5	Female	A	N	N	U
4		VSD-1088	> 5	Male	A	N	N	C
5		VSD-1090	> 5	Female	G	P	N	U
6		VSD-1089	> 5	Female	A	N	N	C

7	Ernakulam, Kerala	VSD-392	≤ 5	Male	Pr	N	N	U
8		VSD-394	> 5	Female	S	N	Y	C
9		VSD-409	≤ 5	Female	A	P	N	U

10	Trivandrum, Kerala	VSD-544	≤ 5	Female	A	N	Y	U
11		VSD-560	≤ 5	Female	S	N	Y	C
12		VSD-562	≤ 5	Male	A	N	N	U
13		VSD-566	> 5	Male	A	N	Y	C
14		VSD-644	≤ 5	Female	G	N	N	U

15	Bhopal, Madhya Pradesh	VSD-886	≤ 5	Male	A	N	N	U
16		VSD-891	≤ 5	Female	A	N	Y	U
17		VSD-894	≤ 5	Female	A	N	N	U
18		VSD-905	≤ 5	Female	G	N	N	U
19		VSD-914	> 5	Male	A	N	N	U

Abbreviations: A, agriculture/animal husbandry; C, caged; G, government; N, no; Pr, private; S, self-employed; U, uncaged; Y, yes.

**Table 3:** Positivity of dog samples for coxiellosis in four selected Indian cities by different diagnostic tests.

Types of samples screened	Total clinical samples screened	Molecular assays		Serological assays
		Trans-PCR (positive)	Com 1-PCR (positive)	IgG-IFAT (positive)
Blood	452	**2**/452	0/452	NA
Serum	452	0/452	0/452	19/452
Vaginal swab	37	0/37	0/37	NA
Uterine tissue	35	0/35	0/35	NA
Total	**976**	**2/452 (0.44%)**	**0 (0%)**	**19/452 (4.20%)**

*Note:* The bold values refer to the total number of samples and per cent.

**Table 4:** Risk factors associated with anti-*C. burnetii* antibodies in dogs in selected cities of India (*χ*^2^ test).

Variables	Categories	Study population	*C. burnetii* IFAT (+)	*C. burnetii* IFAT (−)	Odd's ratio (upper and lower limit)	*p* value	Inference
Gender	Female	168	12	156	2.165 (0.8749–5.574)	0.1056	Ns
	Male	204	7	197			

Contact with stray dog	Yes	42	6	36	4.064 (1.454–11.02)	0.0041	S
	No	330	13	317			

Age	≤ 5 years	231	12	219	1.049 (0.4237–2.706)	0.9220	Ns
	> 5 years	141	7	134			

Caged	No	226	14	212	1.453 (0.5468–3.740)	0.4821	Ns
	Yes	115	5	110			

Dewormed	No	97	5	92	1.013 (0.3926–2.699)	0.9804	Ns
	Yes	275	14	261			

Owners engaged in agriculture and animal husbandry	Yes	165	13	152	2.865 (1.135–7.499)	0.0302	S
	No	207	6	201			

Abbreviations: Ns, nonsignificant; S, significant.

## Data Availability

Data will be made available upon request.

## Supporting Information

Supporting Data 1: Statistical analysis of risk factors. Appendix 1: Questionnaire for the collection of epidemiological data on coxiellosis in dogs.

## References

[1] Asamoah J. K. K., Jin Z., Sun G.-Q., Li M. Y. (2020). A Deterministic Model for Q Fever Transmission Dynamics Within Dairy Cattle Herds: Using Sensitivity Analysis and Optimal Controls. *Computational and Mathematical Methods in Medicine*.

[2] Eldin C., Mélenotte C., Mediannikov O. (2017). From Q Fever to *Coxiella Burnetii* Infection: A Paradigm Change. *Clinical Microbiology Reviews*.

[3] Grace D., Mutua F., Ochungo P. (2012). *Mapping of Poverty and Likely Zoonoses Hotspots*.

[4] Honarmand H. (2012). Q Fever: An Old but Still a Poorly Understood Disease. *Interdisciplinary Perspectives on Infectious Diseases*.

[5] Seshadri R., Paulsen I. T., Eisen J. A. (2003). Complete Genome Sequence of the Q-Fever Pathogen *Coxiella burnetii*. *Proceedings of the National Academy of Sciences*.

[6] Carey K. L., Newton H. J., Lührmann A., Roy C. R. (2011). The *Coxiella burnetii* Dot/Icm System Delivers a Unique Repertoire of Type IV Effectors Into Host Cells and Is Required for Intracellular Replication. *PLoS Pathogens*.

[7] Moos A., Hackstadt T. (1987). Comparative Virulence of Intra- and Interstrain Lipopolysaccharide Variants of *Coxiella Burnetii* in the Guinea Pig Model. *Infection and Immunity*.

[8] Anastácio S., Anjos S., Neves S. (2022). *Coxiella burnetii* in Dogs and Cats From Portugal: Serological and Molecular Analysis. *Pathogens*.

[9] Celina S. S., Cerný J. (2022). *Coxiella burnetii* in Ticks, Livestock, Pets and Wildlife: A Mini-Review. *Frontiers in Veterinary Science*.

[10] Lakshmin D. (2020). The New Cool: Adopting Street Dogs Is Gaining Popularity in India. *National Geographic Newsletter*.

[11] Ma G. C., Norris J. M., Mathews K. O. (2020). New Insights on the Epidemiology of *Coxiella burnetii* in Pet Dogs and Cats From New South Wales, Australia. *Acta Tropica*.

[12] Ebani V. V. (2020). Retrospective Study on the Occurrence of Antibodies Against *Coxiella burnetii* in Dogs From Central Italy. *Pathogens*.

[13] Sheikh N., Kumar S., Sharma H. K., Bhagyawant S. S., Thavaselvam D. (2020). Development of a Rapid and Sensitive Colorimetric Loop-Mediated Isothermal Amplification Assay: A Novel Technology for the Detection of *Coxiella burnetii* From Minimally Processed Clinical Samples. *Frontiers in Cellular and Infection Microbiology*.

[14] Berri M., Laroucau K., Rodolakis A. (2000). The Detection of *Coxiella burnetii* From Ovine Genital Swabs, Milk and Fecal Samples by the Use of a Single Touchdown Polymerase Chain Reaction. *Veterinary Microbiology*.

[15] Zhang G. Q., Hotta A., Mizutani M. (1998). Direct Identification of *Coxiella burnetii* Plasmids in Human Sera by Nested PCR. *Journal of Clinical Microbiology*.

[16] Tukur S. M., Mohammed K., Watanabe M., Rani P. A. M. A., Watanabe M. (2019). *Coxiella burnetii* Detection in Stray Dogs in Klang Valley, Malaysia. *Journal of Advances in Microbiology*.

[17] Roest H. I. J., van Solt C. B., Tilburg J. J. H. C. (2013). Search for Possible Additional Reservoirs for Human Q Fever, the Netherlands. *Emerging Infectious Diseases*.

[18] Roest H. I. J., Bossers A., van Zijderveld F. G., Rebel J. M. L. (2013). Clinical Microbiology of *Coxiella burnetii* and Relevant Aspects for the Diagnosis and Control of the Zoonotic Disease Q Fever. *Veterinary Quarterly*.

[19] S El-Mahallawy H., Kelly P., Zhang J. (2015). Serological and Molecular Evidence of *Coxiella burnetii* in Samples From Humans and Animals in China. *Annals of Agricultural and Environmental Medicine*.

[20] De Majo M., Donato G., Masucci M. (2021). Bidimensional and Contrast-Enhanced Ultrasonography of the Spleen in Dogs Affected by Leishmaniosis. *Animals*.

[21] Iatta R., Sazmand A., Nguyen V.-L. (2021). Vector-Borne Pathogens in Dogs of Different Regions of Iran and Pakistan. *Parasitology Research*.

[22] Abdel-Moein K. A., Zaher H. M. (2021). Parturient Cat as a Potential Reservoir for *Coxiella burnetii*: A Hidden Threat to Pet Owners. *Vector Borne and Zoonotic Diseases*.

[23] Huggins L. G., Colella V., Koehler A. V., Schunack B., Traub R. J. (2022). A Multipronged Next‐Generation Sequencing Metabarcoding Approach Unearths Hyperdiverse and Abundant Dog Pathogen Communities in Cambodia. *Transboundary and Emerging Diseases*.

[24] Chitanga S., Simulundu E., Simuunza M. C. (2018). First Molecular Detection and Genetic Characterization of *Coxiella burnetii* in Zambian Dogs and Rodents. *Parasites and Vectors*.

[25] Rezaei M., Rezaei M., Khalili M., Akhtardanesh B., Shahheidaripour S. (2016). Q Fever in Dogs: An Emerging Infectious Disease in Iran. *Journal of Medical Bacteriology*.

[26] Patra G., Ghosh S., Priyanka E. M. A. (2020). Molecular Detection of *Coxiella burnetii* and *Borrelia Burgdorferi* in Ticks Infesting Goats in North-Eastern States of India. *International Journal of Acarology*.

[27] Plummer P. J., McClure J. T., Menzies P., Morley P. S., Van den Brom R., Van Metre D. C. (2018). Management of *Coxiella burnetii* Infection in Livestock Populations and the Associated Zoonotic Risk: A Consensus Statement. *Journal of Veterinary Internal Medicine*.

[28] Turcotte M., Denis-Robichaud J., Dubuc J. (2021). Prevalence of Shedding and Antibody to *Coxiella burnetii* in Post-Partum Dairy Cows and Its Association With Reproductive Tract Diseases and Performance: A Pilot Study. *Preventive Veterinary Medicine*.

[29] Kidd L. (2019). *Small Animal Internal Medicine*.

[30] Boni M., Davoust B., Tissot-Dupont H., Raoult D. (1998). Survey of Seroprevalence of Q Fever in Dogs in the Southeast of France, French Guyana, Martinique, Senegal and the Ivory Coast. *Veterinary Microbiology*.

[31] Havas K. A., Burkman K. (2011). A Comparison of the Serological Evidence of *Coxiella burnetii* Exposure Between Military Working Dogs and Feral Canines in Iraq. *Military Medicine*.

[32] Shapiro A. J., Norris J. M., Heller J., Brown G., Malik R., Bosward K. L. (2016). Seroprevalence of *Coxiella burnetii* in Australian Dogs. *Zoonoses and Public Health*.

[33] Lyoo K.-S., Kim D., Jang H. G., Lee S.-J., Park M. Y., Hahn T.-W. (2017). Prevalence of Antibodies against *Coxiella burnetii* in Korean Native Cattle, Dairy Cattle, and Dogs in South Korea. *Vector Borne and Zoonotic Diseases*.

[34] Stefanetti V., Compagnone A., Sordini C. (2018). Retrospective Biomolecular Investigation of *Coxiella burnetii* and Leptospira Spp. DNA in Cases of Abortion, Stillbirth and Neonatal Mortality in Dogs and Cats. *Topics in Companion Animal Medicine*.

[35] Marrie T. J., Van Buren J., Fraser J. (1985). Seroepidemiology of Q Fever Among Domestic Animals in Nova Scotia. *American Journal of Public Health*.

[36] Faix D. J., Harrison D. J., Riddle M. S. (2008). Outbreak of Q Fever Among US Military in Western Iraq, June–July 2005. *Clinical Infectious Diseases*.

[37] Kelly P. J., Matthewman L. A., Mason P. R., Raoult D. (1993). Q Fever in Zimbabwe. A Review of the Disease and the Results of a Sero Survey of Humans, Cattle, Goats and Dogs. *South African Medical Journal*.

[38] Htwe K., Amano K., Sugiyama Y. (1992). Seroepidemiology of *Coxiella burnetii* in Domestic and Companion Animals in Japan. *The Veterinary Record*.

[39] Wardrop N. A., Thomas L. F., Cook E. A. J. (2016). The Sero-Epidemiology of *Coxiella burnetii* in Humans and Cattle, Western Kenya: Evidence From a Cross-Sectional Study. *PLoS Neglected Tropical Diseases*.

[40] Proboste T., Deressa F. B., Li Y., Kal D. O., Gelalcha B. D., Soares Magalhães R. J. (2021). Geographical Variation in *Coxiella burnetii* Seroprevalence in Dairy Farms Located in South-Western Ethiopia: Understanding the Broader Community Risk. *Pathogens*.

[41] Kirchgessner M. S., Dubovi E. J., Whipps C. M. (2013). Disease Risk Surface for *Coxiella burnetii* Seroprevalence in White‐Tailed Deer. *Zoonoses and Public Health*.

[42] Kumar M., Singh Malik S., Ramanjeneya S. (2019). A Cross-Sectional Study on the Occurrence of *Coxiella burnetii* Infection in a Dairy Farm, Bareilly, India. *International Journal of Current Microbiology and Applied Sciences*.

[43] Balamurugan V., Alamuri A., Kumar K. V., Govindaraj G., Roy P. (2021). Prevalence of *Coxiella burnetii* Antibodies in Dairy Cattle Associated With Abortions and Reproductive Disorders. *Proceedings of the National Academy of Sciences, India—Section B: Biological Sciences*.

[44] Amin F., Ali S., Javid A. (2022). Sero-Epidemiology of *Coxiella burnetii* Infection in Small Ruminants in the Eastern Region of Punjab, Pakistan. *Pathogens*.

[45] Esmailnejad A., Abbaszadeh Hasiri M. (2017). Serological Evidence of *Coxiella burnetii* Infection Among Companion Dogs in Fars Province, South Iran. *Bulgarian Journal of Veterinary Medicine*.

[46] Howard Z. P., Omsland A. (2020). Selective Inhibition of Coxiella Burnetii Replication by the Steroid Hormone Progesterone. *Infection and Immunity*.

