# Supplementary material for: Coxiellosis in Dogs—A Hitherto Masked Zoonosis in India: An Insight From Seromolecular Investigation and Risk Factor Analysis
Source: Can J Infect Dis Med Microbiol. 2025 Jan 20;2025:8642619. doi: 10.1155/cjid/8642619 (PMC11772063; doi:10.1155/cjid/8642619)
Supplement: Supporting Information — Additional supporting information can be found online in the Supporting Information section. [file 8642619.f1.docx]

**Supplementary Data 1.**  **Statistical analysis of risk factors**

| 1. **Table Analyzed** | **Sex** |  |  |
| --- | --- | --- | --- |
|  |  |  |  |
| P value and statistical significance |  |  |  |
| Test | Chi-square |  |  |
| Chi-square, df | 2.618, 1 |  |  |
| z | 1.618 |  |  |
| P value | 0.1056 |  |  |
| P value summary | ns |  |  |
| One- or two-sided | Two-sided |  |  |
| Statistically significant (P < 0.05)? | No |  |  |
|  |  |  |  |
| Effect size | Value | 95% CI |  |
| Odds ratio | 2.165 | 0.8749 to 5.574 |  |
| Reciprocal of odds ratio | 0.4619 | 0.1794 to 1.143 |  |
|  |  |  |  |
| Methods used to compute CIs |  |  |  |
| Odds ratio | Baptista-Pike |  |  |
|  |  |  |  |
| Data analyzed | IFAT POSITIVE | IFAT NEGATIVE | Total |
| Female | 12 | 156 | 168 |
| male | 7 | 197 | 204 |
| Total | 19 | 353 | 372 |
|  |  |  |  |
| Percentage of row total | IFAT POSITIVE | IFAT NEGATIVE |  |
| Female | 7.14% | 92.86% |  |
| male | 3.43% | 96.57% |  |
|  |  |  |  |
| Percentage of column total | IFAT POSITIVE | IFAT NEGATIVE |  |
| Female | 63.16% | 44.19% |  |
| male | 36.84% | 55.81% |  |
|  |  |  |  |
| Percentage of grand total | IFAT POSITIVE | IFAT NEGATIVE |  |
| Female | 3.23% | 41.94% |  |
| male | 1.88% | 52.96% |  |

| 1. **Table Analyzed** | **Contact with stray dogs** |  |  |
| --- | --- | --- | --- |
|  |  |  |  |
| P value and statistical significance |  |  |  |
| Test | Chi-square |  |  |
| Chi-square, df | 8.229, 1 |  |  |
| z | 2.869 |  |  |
| P value | 0.0041 |  |  |
| P value summary | ** |  |  |
| One- or two-sided | Two-sided |  |  |
| Statistically significant (P < 0.05)? | Yes |  |  |
|  |  |  |  |
| Effect size | Value | 95% CI |  |
| Odds ratio | 4.064 | 1.454 to 11.02 |  |
| Reciprocal of odds ratio | 0.2461 | 0.09071 to 0.6878 |  |
|  |  |  |  |
| Methods used to compute CIs |  |  |  |
| Odds ratio | Baptista-Pike |  |  |
|  |  |  |  |
| Data analyzed | IFAT POSITIVE | IFAT NEGATIVE | Total |
| Stray contact | 6 | 36 | 42 |
| No stray contact | 13 | 317 | 330 |
| Total | 19 | 353 | 372 |
|  |  |  |  |
| Percentage of row total | IFAT POSITIVE | IFAT NEGATIVE |  |
| Stray contact | 14.29% | 85.71% |  |
| No stray contact | 3.94% | 96.06% |  |
|  |  |  |  |
| Percentage of column total | IFAT POSITIVE | IFAT NEGATIVE |  |
| Stray contact | 31.58% | 10.20% |  |
| No stray contact | 68.42% | 89.80% |  |
|  |  |  |  |
| Percentage of grand total | IFAT POSITIVE | IFAT NEGATIVE |  |
| Stray contact | 1.61% | 9.68% |  |
| No stray contact | 3.49% | 85.22% |  |

| 1. **Table Analyzed** | **Age** |  |  |
| --- | --- | --- | --- |
|  |  |  |  |
| P value and statistical significance |  |  |  |
| Test | Chi-square |  |  |
| Chi-square, df | 0.009579, 1 |  |  |
| z | 0.09787 |  |  |
| P value | 0.9220 |  |  |
| P value summary | ns |  |  |
| One- or two-sided | Two-sided |  |  |
| Statistically significant (P < 0.05)? | No |  |  |
|  |  |  |  |
| Effect size | Value | 95% CI |  |
| Odds ratio | 1.049 | 0.4237 to 2.706 |  |
| Reciprocal of odds ratio | 0.9534 | 0.3696 to 2.360 |  |
|  |  |  |  |
| Methods used to compute CIs |  |  |  |
| Odds ratio | Baptista-Pike |  |  |
|  |  |  |  |
| Data analyzed | IFAT POSITIVE | IFAT NEGATIVE | Total |
| 5 years or less | 12 | 219 | 231 |
| more than 5 yrs | 7 | 134 | 141 |
| Total | 19 | 353 | 372 |
|  |  |  |  |
| Percentage of row total | IFAT POSITIVE | IFAT NEGATIVE |  |
| 5 years or less | 5.19% | 94.81% |  |
| more than 5 yrs | 4.96% | 95.04% |  |
|  |  |  |  |
| Percentage of column total | IFAT POSITIVE | IFAT NEGATIVE |  |
| 5 years or less | 63.16% | 62.04% |  |
| more than 5 yrs | 36.84% | 37.96% |  |
|  |  |  |  |
| Percentage of grand total | IFAT POSITIVE | IFAT NEGATIVE |  |
| 5 years or less | 3.23% | 58.87% |  |
| more than 5 yrs | 1.88% | 36.02% |  |

| 1. **Table Analyzed** | **Type of housing** |  |  |
| --- | --- | --- | --- |
|  |  |  |  |
| P value and statistical significance |  |  |  |
| Test | Chi-square |  |  |
| Chi-square, df | 0.4941, 1 |  |  |
| z | 0.7029 |  |  |
| P value | 0.4821 |  |  |
| P value summary | ns |  |  |
| One- or two-sided | Two-sided |  |  |
| Statistically significant (P < 0.05)? | No |  |  |
|  |  |  |  |
| Effect size | Value | 95% CI |  |
| Odds ratio | 1.453 | 0.5468 to 3.740 |  |
| Reciprocal of odds ratio | 0.6883 | 0.2674 to 1.829 |  |
|  |  |  |  |
| Methods used to compute CIs |  |  |  |
| Odds ratio | Baptista-Pike |  |  |
|  |  |  |  |
| Data analyzed | IFAT POSITIVE | IFAT NEGATIVE | Total |
| No cage | 14 | 212 | 226 |
| cage | 5 | 110 | 115 |
| Total | 19 | 322 | 341 |
|  |  |  |  |
| Percentage of row total | IFAT POSITIVE | IFAT NEGATIVE |  |
| No cage | 6.19% | 93.81% |  |
| cage | 4.35% | 95.65% |  |
|  |  |  |  |
| Percentage of column total | IFAT POSITIVE | IFAT NEGATIVE |  |
| No cage | 73.68% | 65.84% |  |
| cage | 26.32% | 34.16% |  |

| 1. **Table Analyzed** | **Deworming** |  |  |
| --- | --- | --- | --- |
|  |  |  |  |
| P value and statistical significance |  |  |  |
| Test | Chi-square |  |  |
| Chi-square, df | 0.0006009, 1 |  |  |
| z | 0.02451 |  |  |
| P value | 0.9804 |  |  |
| P value summary | ns |  |  |
| One- or two-sided | Two-sided |  |  |
| Statistically significant (P < 0.05)? | No |  |  |
|  |  |  |  |
| Effect size | Value | 95% CI |  |
| Odds ratio | 1.013 | 0.3926 to 2.699 |  |
| Reciprocal of odds ratio | 0.9870 | 0.3705 to 2.547 |  |
|  |  |  |  |
| Methods used to compute CIs |  |  |  |
| Odds ratio | Baptista-Pike |  |  |
|  |  |  |  |
| Data analyzed | IFAT POSITIVE | IFAT NEGATIVE | Total |
| Not dewormed | 5 | 92 | 97 |
| Dewormed | 14 | 261 | 275 |
| Total | 19 | 353 | 372 |
|  |  |  |  |
| Percentage of row total | IFAT POSITIVE | IFAT NEGATIVE |  |
| Not dewormed | 5.15% | 94.85% |  |
| Dewormed | 5.09% | 94.91% |  |
|  |  |  |  |
| Percentage of column total | IFAT POSITIVE | IFAT NEGATIVE |  |
| Not dewormed | 26.32% | 26.06% |  |
| Dewormed | 73.68% | 73.94% |  |
|  |  |  |  |
| Percentage of grand total | IFAT POSITIVE | IFAT NEGATIVE |  |
| Not dewormed | 1.34% | 24.73% |  |
| Dewormed | 3.76% | 70.16% |  |

| 1. **Table Analyzed** | **Nature of occupation** |  |  |
| --- | --- | --- | --- |
|  |  |  |  |
| P value and statistical significance |  |  |  |
| Test | Chi-square |  |  |
| Chi-square, df | 4.699, 1 |  |  |
| z | 2.168 |  |  |
| P value | 0.0302 |  |  |
| P value summary | * |  |  |
| One- or two-sided | Two-sided |  |  |
| Statistically significant (P < 0.05)? | Yes |  |  |
|  |  |  |  |
| Effect size | Value | 95% CI |  |
| Odds ratio | 2.865 | 1.135 to 7.499 |  |
| Reciprocal of odds ratio | 0.3490 | 0.1334 to 0.8809 |  |
|  |  |  |  |
| Methods used to compute CIs |  |  |  |
| Odds ratio | Baptista-Pike |  |  |
|  |  |  |  |
| Data analyzed | IFAT POSITIVE | IFAT NEGATIVE | Total |
| agriculture and animal husbandry | 13 | 152 | 165 |
| Other occupation | 6 | 201 | 207 |
| Total | 19 | 353 | 372 |
|  |  |  |  |
| Percentage of row total | IFAT POSITIVE | IFAT NEGATIVE |  |
| agriculture and animal husbandry | 7.88% | 92.12% |  |
| Other occupation | 2.90% | 97.10% |  |
|  |  |  |  |
| Percentage of column total | IFAT POSITIVE | IFAT NEGATIVE |  |
| agriculture and animal husbandry | 68.42% | 43.06% |  |
| Other occupation | 31.58% | 56.94% |  |
|  |  |  |  |
| Percentage of grand total | IFAT POSITIVE | IFAT NEGATIVE |  |
| agriculture and animal husbandry | 3.49% | 40.86% |  |
| Other occupation | 1.61% | 54.03% |  |

**Appendix 1. Questionnaire for the collection of epidemiological data on coxiellosis in dogs**

| **Parameters** | **Response** |  |
| --- | --- | --- |
| **Details of Pet owner** |  |  |
| Name & Place; Phone number |  |  |
| Occupation |  |  |
| Total number of pet animals owned |  |  |
| Categories and nos. of livestock species owned | Cattle/Buffalo /Sheep/Goat/ Poultry/Cat |  |
| Occupational exposure to livestock | Yes/No |  |
| Are you aware of the zoonotic disease-Q fever? | Yes/No |  |
| Any coxiellosis-associated symptom(s) noticed among family member(s) If yes, what are they? | Yes/No  Spontaneous abortion mainly in first trimester / Pyrexia of Unknown Origin (PUO)/ chronic fatigue syndrome (CFS)/ Infectious Endocarditis / Chronic hepatitis/ Osteomyelitis/ Osteoarthritis/ Pneumonitis / Spontaneous meningitis |  |
| **Details of pet animals presented in clinics** |  |  |
| Breed |  |  |
| Age | (<1year, 1-5 years, >5 years |  |
| Sex | Male/Female |  |
| Castrated /Spayed | Yes/No |  |
| Body Condition Score | Underweight/ ideal/ overweight |  |
| Diet | Commercial dog food (Wet)/ Commercial dog food (Dry)/ Raw meat / Homemade food /Others……………… |  |
| The presence of Fleas or Ticks on pets noticed by Physical Examination | Yes/No |  |
| Is your dog confined to a yard? (by a fence or by chain) | Yes/No/NA |  |
| Housing | Cage/ free in the yard/ Both practised |  |
| Frequency of cleaning cages | Daily/ Weekly/ Monthly/ Specify |  |
| Floor disinfection practised and its frequency | Yes/No |  |
| Hand disinfection practised and its frequency | Yes/No |  |
| Does your pet come in contact (directly or indirectly) with stray dogs? If yes, then how frequently? | Yes/No |  |
| Does your pet come in contact (directly or indirectly) with livestock (sheep, goats, cattle)? If yes, then how frequently? | Yes/No |  |
| If Yes, with what species does the dog have contact? |  |  |
| How did the dog come in contact? | On your property / on a neighbouring property/ on walks/ on Agricultural fields |  |
| **Reproduction history** |  |  |
| **Treatment history** | Type of Antibiotic used  Duration of antibiotic therapy |  |
| History of Abortions/ Repeat breeding/ Stillbirth/ Anestrous | Observed/Not observed; Frequency ………..  Number……….. |  |
| **Disposal of wastes** |  |  |
| Place of disposal | Within the courtyard/ Outside the courtyard/Municipal waste disposal |  |
| Method of biological waste and excreta disposal | Burial/Open surface /Using Disinfectant |  |
